# ‘Why Don't We Get Counselling?’: Comparing NICE Guidelines for Morphological and Genetic Cancer Risk Diagnoses

**DOI:** 10.1002/cam4.70607

**Published:** 2025-01-15

**Authors:** Elspeth Davies

**Affiliations:** ^1^ Nuffield Department of Primary Care Health Sciences University of Oxford Oxford UK

## Abstract

**Background:**

In the UK's National Health Service (NHS), there is specific psychosocial care offered to people with genetic cancer risk conditions but not morphological cancer risk conditions. As researchers develop new ways to diagnose morphological risk conditions, including precancers and in situ cancers, it is important to consider the psychosocial care that those diagnosed might require.

**Objectives:**

This study compares the National Institute for Health and Care Excellence's guidelines for BRCA1/2, which are genetic risk conditions, and Barrett's oesophagus (BO), a morphological risk condition. It then theorises reasons for the similarities and differences made visible by this comparative work.

**Methods:**

The author completed an in‐depth analysis of two sets of NICE guidelines, before carrying out a review of historical and social scientific literature on cancer risk to offer potential explanations for the disparities identified.

**Results:**

The ‘right not to know’ is protected in the case of BRCA1/2 diagnoses, but not BO. Additionally, specialist counselling is required for people receiving diagnoses of genetic risk but not offered for those diagnosed with morphological risk conditions. The paper offers four possible reasons for these disparities, concluding that they appear to be in large part due to historic genetic exceptionalism, rather than differences in patients' needs.

**Conclusion:**

There may be a need to consider offering further psychosocial care to people with morphological risk conditions like BO. Lessons might be learnt from the field of genetic counselling.

Although once focused primarily on the treatment of people with symptoms, clinical practice in the UK's National Health Service (NHS), as well as in other well‐funded healthcare systems, is becoming increasingly aimed at diagnosing and managing statistical risk of future disease. This is particularly the case in the field of oncology, which has seen a significant shift in recent years towards prevention and early detection through measures that seek to identify and reduce cancer risk [1]. This essay focuses on one specific and growing area of this broader shift: the diagnosis of genetic and morphological (meaning diagnosed on the basis of abnormalities in the structure of tissues and cells) cancer risk diagnoses.

Both types of diagnosis pursue similar goals, searching for abnormalities that enable risk diagnosis and risk management [2]. Patient experiences can also be comparable, with both types of diagnosis sometimes offering reassurance as well as creating anxiety and uncertainty regarding whether cancer will develop in the future [3, 4]. Nevertheless, despite these similarities, the care recommended to people living with genetic and morphological diagnoses by the NHS differs significantly. This essay seeks to understand this disparity in more detail using BRCA1/2, genetic risk diagnoses, and Barrett's oesophagus (BO), a morphological risk diagnosis, as case studies.

The need for this work was realised during the author's doctoral research on patient experiences of BO. One patient had asked during an interview: ‘why don't we get counselling?’ while discussing how difficult she had found her BO diagnosis and how little psychosocial care she felt had been offered on the NHS. This essay seeks to offer answers to this patient's excellent question. More generally, it highlights potential oversights in care provision as medicine shifts towards becoming increasingly risk‐centred, both in the UK and elsewhere.

## 1 Background

BO is a condition in which the normal squamous epithelium lining of the oesophagus is replaced by intestinalized columnar epithelium. It is a known risk factor or precursor for oesophageal adenocarcinoma (OAC). Unlike some other morphological risk conditions such as colon polyps or melanoma in situ, BO is usually not removed. Instead, those diagnosed are offered regular and sometimes lifelong endoscopic surveillance.

BO is usually detected during an upper GI endoscopy and diagnosed following histopathological examination of biopsies. Eligibility for routine or urgent upper GI endoscopy is largely determined by symptoms, such as dyspepsia, persistent vomiting, dysphagia or unexplained weight loss, as well as risk factors such as age.

BRCA1 and BRCA2 are genes that produce proteins that help repair damaged DNA. Harmful inherited variants of these genes can cause an increased risk of several types of cancer, particularly breast and ovarian. BRCA1/2 mutations are identified via genetic testing, a practice defined as the “analysis of human DNA, RNA, chromosomes, proteins, and certain metabolites in order to detect heritable disease‐related genotypes, mutations, phenotypes, or karyotypes for clinical purposes. Such purposes include predicting risk of disease, identifying carriers, establishing prenatal and clinical diagnosis or prognosis.” [5] Patients diagnosed with BRCA1/2 mutations may be offered annual breast screening, chemoprevention medications, risk‐reducing surgeries, as well as being encouraged to carry out self‐examinations and to make lifestyle changes.

Eligibility for BRCA1/2 genetic testing is currently determined on the basis of risk rather than symptoms: referrals are most commonly made on the basis of personal or family histories of breast cancer.

Both BO and BRCA1/2 mutation diagnoses are diagnosed with the aim of facilitating cancer early detection and improving survival rates. Some patients may find risk diagnosis and management reassuring, while for others these practices can cause anxiety and lead to a decrease in health‐related quality of life (HRQL) [6, 7, 8].

## 2 Methods

This essay explores disparities in the National Institute for Health and Care Excellence (NICE) [9] guidelines concerning care provision for people living with different types of cancer risk [10]. The author speculates reasons for these disparities with reference to social scientific and historical literature.

## 3 Results

### 3.1 Diagnostic Pathways

Diagnostic pathways for BO and BRCA1/2 are notably different. BO is diagnosed during routine or urgent endoscopies. Patients are usually primarily referred for endoscopies on the basis of their symptoms. Concerns regarding BO are only one reason for endoscopic investigation. Unless they are undergoing proactive case finding for BO [11], patients are not routinely informed about the possibility of diagnosing BO prior to the procedure.

In contrast, BRCA1/2 requires specific genetic testing. Pre‐test counselling (preferably two sessions) is required, in part to ensure that patients understand and consent to the potential outcomes of this testing.

In other words, people explicitly consent to the possibility of being diagnosed with a BRCA1/2 abnormality prior to genetic testing. They do not explicitly consent to the possibility of being diagnosed with BO prior to an endoscopy.

The ‘right not to know’ is exercised and protected in the case of BRCA1/2 abnormalities but not BO. In part, this is because the genetic test for BRCA1/2 is only looking for these specific conditions, while an endoscopy can lead to a multitude of different diagnoses, including BO. Although patients do not have the right not to know about BO, they do have the right to opt out of surveillance programmes following their diagnosis. These decisions should be made following conversations about the pros and cons of surveillance with their clinicians.

### 3.2 Psychosocial Care

After a BO diagnosis, patients are offered a clinical consultation to discuss cancer risk, surveillance plans and symptom control. They should also be provided with information of patient support groups. No specialist psychosocial counselling is available for patients prior to or following a diagnosis of BO.

Genetic counselling is provided to people who meet the criteria for BRCA1/2 testing because it is linked to reduced anxiety and cancer concerns, as well as enhanced accuracy regarding perceived risk in the short term. Even those who do not meet the criteria for referral to secondary care can be referred to specialist counselling.

Note that this care provision may change with recent shifts towards introducing population‐based genetic testing for BRCA1/2 and other variants related to cancer risk [12]. Such developments may remove the requirements for clinical consultation prior to genetic testing that have long existed in this field. For now, however, genetic counselling remains an important part of BRCA1/2 testing pathways.

## 4 Discussion

This section considers four possible explanations for these disparities.

### 4.1 BRCA1/2 Mutations Pose Higher Lifetime Cancer Risk than BO

People with BRCA1/2 mutations are more at risk of breast cancer than people with BO are at risk of OAC. By the time they are 70, a person with a BRCA1 mutation has a 65%–85% risk of developing breast cancer. This risk is 40%–80% for BRCA2 mutation carriers [13]. In contrast, the lifetime risk of developing OAC following a diagnosis of BO is 7%–12.5% according to recent meta‐analyses and 2.7%–10% based on data from recent population‐based studies [14]. Perhaps this greater risk might explain the provision of counselling for people with BRCA1/2 mutations but not BO.

Nevertheless, studies find that people with BRCA1/2 and BO often wrongly estimate their cancer risk [15, 16]. Research repeatedly concludes that statistical risk is not correlated to levels of anxiety experienced, for example [15]. Thus, this difference in statistical risk alone does not explain the disparities in care: psychosocial interventions can be beneficial regardless of objective cancer risk.

### 4.2 Genetic Diagnoses Have Particular Consequences for Relatives

It may be hypothesised that specialist counselling is required for people with genetic risk diagnoses in particular because of the consequences that these diagnoses have for patients' relatives. However, research suggests that people with BO also sometimes worry greatly about their family members' risk of OAC and have concerns surrounding disclosing their diagnosis [17]. Both genetic and morphological risk diagnoses can have complex consequences for patients' relatives; this does not sufficiently explain why people with one form of diagnosis received specialist psychosocial support but not the other.

### 4.3 BRCA1/2 Abnormalities Are Asymptomatic; BO Is Associated with Symptoms

Although BO is thought to asymptomatic in itself, it has become associated with symptoms of acid reflux because it is considered to be a complication of gastro‐oesophageal reflux disease (GORD) [18]. GORD is a chronic condition in which stomach acid leaks into the oesophagus, causing symptoms such a heartburn and oesophagitis. BO can also affect people without these symptoms, although they are not commonly diagnosed because at present asymptomatic people are not offered endoscopic investigation.

Symptom management is an important part of the care offered to people with BO, particularly because BO patients' anxiety regarding future cancers tends to be worse when acid reflux symptoms are uncontrolled [4]. Advice regarding symptom management is offered in clinical settings. It may be assumed that patients have also had the chance to discuss potential worries regarding cancer risk in these consultations.

In contrast, BRCA1/2 abnormalities are asymptomatic, meaning that there is a clearer need to provide a different type of care to the interventions that have traditionally been offered in the clinic aimed at treating symptoms. Genetic counselling was developed to meet this need. The relevance of specialised psychosocial interventions for people with BO may be less immediately obvious because of the assumption that fear can be addressed in standard consultations. The significant worry experienced by patients joining growing large support groups for people with BO, particularly online, suggests these consultations may not always be sufficiently reassuring [17].

### 4.4 Historic Genetic Exceptionalism

Historian Ilana Löwy convincingly argues that the disparity in care described in this article can be explained by the novelty of genetic testing when it was invented. This new technology was deemed to present unique ethical dilemmas, leading to a huge amount of research into the ‘psychosocial’ effects of genetic testing [19]. Genetic risk diagnoses have not been assumed to be self‐evidently valuable in the same way as morphological risk conditions.

The pathological laboratory examination and endoscopy technologies used to diagnose BO have existed for longer than genetic testing, although new developments have changed some of the practices involved [20, 21]. The development of these older technologies has not seemed to warrant the same extensive ethical consideration as the introduction of new ones. Since the 1990s, this the idea that genetic information is unique and different from other kinds of medical information has sometimes been described as ‘genetic exceptionalism’ [22].

The differences in attention to psychosocial care in genetic and morphological cancer risk conditions appear to be the result of historical differences rather than a lack of need.

## 5 Clinical Implications

This work suggests that, if it is offered to patients with genetic risk conditions, perhaps pre‐ and post‐diagnostic counselling should also be considered in the case of morphological cancer risk conditions. Drawing on evidence supporting genetic counselling, it is possible to hypothesise that the provision of specialist psychosocial care for people with BO might help reduce anxiety and manage concerns about cancer, as well as increasing risk accuracy and knowledge.

## 6 Study Limitations

This study offers a comparison of two NICE guidelines and then uses existing literature to speculatively explore reasons for these disparities. It also suggests possible changes that could be made to improve clinical care for people with BO. Further research is needed to validate these suggestions for both BO and other morphological risk diagnoses.

## 7 Conclusion

Let us return to the insightful question that inspired this research: why are Barrett's oesophagus patients not offered specialist counselling? This paper has outlined the disparities in care underlying this question in more detail by comparing the NICE guidelines for people with BO and BRCA1/2. It has suggested that a relative lack of psychosocial care for people with BO appears to have emerged due to the association of BO with symptoms but not BRCA1/2, as well as the varying historical developments of different types of testing. As researchers and clinicians increasingly discuss screening for BO in the UK and elsewhere, we should consider the care that might be necessary as increasing numbers of people are transformed into patients on the basis of cancer risk diagnoses [23]. The question that remains, however, is how such an intervention might be offered in the context of a health service that is increasingly becoming risk‐centred amidst severe resource scarcity.

## Author Contributions

**Elspeth Davies:** conceptualization (lead), formal analysis (lead), investigation (lead), methodology (lead), project administration (lead), writing – original draft (lead), writing – review and editing (lead).

## Ethics Statement

The author has nothing to report.

## Consent

The author has nothing to report.

## Conflicts of Interest

The author declares no conflicts of interest.

## Data Availability

Data sharing is not applicable to this article as no new data were created or analyzed in this study.
